# The Influence of Internet Usage Frequency on Women’s Fertility Intentions—The Mediating Effects of Gender Role Attitudes

**DOI:** 10.3390/ijerph18094784

**Published:** 2021-04-30

**Authors:** Pengcheng Liu, Jingjing Cao, Wenjie Nie, Xiaojie Wang, Yani Tian, Cheng Ma

**Affiliations:** 1 School of Economics, Qingdao University, Qingdao 266061, China; qd_lpc@qdu.edu.cn; 2 School of Business, Qingdao University, Qingdao 266061, China; 2018020946@qdu.edu.cn (J.C.); 2013910056@qdu.edu.cn (C.M.); 3 School of Management, Ocean University of China, Qingdao 266100, China; niewenjie@stu.ouc.edu.cn; 4 Business School, The University of Sydney, Sydney, NSW 2006, Australia; ytia4093@uni.sydney.edu.au

**Keywords:** internet usage, fertility intention, gender role attitudes, China

## Abstract

The purpose of this study is to verify the influence of internet usage frequency on women’s fertility intentions and to examine the mediating effects of gender role attitudes, under the influence of internet usage frequency that affects women’s fertility intentions, combined with the specific Chinese cultural context. A cross-sectional secondary data analysis was conducted using a sample of 3113 women of childbearing age in the Chinese General Social Survey in 2017 (CGSS2017). The results of the negative binomial regression model showed that, under the premise of controlling individual characteristic variables, the higher the frequency of internet usage, the lower the fertility intention (*p* < 0.01). The results of the mediating effect model show that the more frequently women use the internet, the lower their fertility intentions, and the less they agree with Chinese traditional gender roles, which are “men work outside to support the family while women stay at home to take care of the family”. These findings have implications in formulating public policies aimed at increasing the fertility rate; that is, it is not enough to increase women’s fertility intentions under China’s universal two-child policy. Moreover, public policy formulators need to consider gender role attitudes and the influence of the internet as a method for dissemination of information.

## 1. Introduction

The implementation of the Chinese universal two-child policy is seen as an important measure to ease the pressures of a reducing labor force and population aging [1]. However, in terms of the actual effects of this policy, the fertility potential released by China’s fertility policy adjustment is far from expected. The influences of non-policy factors on women’s fertility intentions, and fertility behaviors, have gradually become apparent [2]. Since the 1990s, the rapid expansion of the internet has characterized a digital revolution, creating widespread social implications [3]. The internet has become the most important means for people to communicate, profoundly affecting individual lifestyles and behavioral decisions [4,5,6,7,8], including women’s fertility intentions. 

These profound social and economic implications, as a result of the internet, have been underlined by social science scholars throughout the years [3]. Related empirical studies focused on the effects of the internet on marriage rates [9], teenage fertility [10], and the labor force participation of married women [11,12,13]. For example, Guldi and Herbst [10] studied the impacts of broadband access on the fertility decision-makings of American adolescents, and found that an increase in internet broadband access was associated with a reduction in the adolescent birth rate. Francesco [11] used German panel data and found that the internet can effectively alleviate work–family conflicts by increasing the possibility of remote work, thereby creating a positive impact on women’s fertility intentions. However, few related studies have specifically explored the relationship between internet usage frequency and individual fertility intentions in the Chinese social–cultural context. This is the subject of our research.

As a pervasive innovation that affects all realms of society, internet usage likely affects female fertility intentions. On the one hand, internet usage has reduced the cost of accessing information about contraceptives, and the possible consequences of choosing whether or not to become a parent (e.g., birth risks and postpartum complications, anecdotal evidence on parenting, and the costs and benefits of raising a child) [11]. On the other hand, the openness of the internet and its interactive characteristics have changed the traditional ways of forming partnerships and reshaped the state of communication among neighbors, colleagues, and friends [2,14,15,16]. In contrast to traditional one-directional media, the internet may enhance social interactions, regarded as an important channel that affects fertility decisions, via social learning, social influence, or both. Internet usage may also decrease the amount of time spent with a partner (or searching for a partner) and, consequently, can negatively affect one’s fertility intentions [11].

In addition to the direct effects, the effects of internet usage on women’s fertility intentions can be moderated by gender roles in specific social and cultural contexts in China. China’s traditional concepts of gender roles can imprison women in families, who are responsible for housework and child rearing. By these concepts, fertility intentions are generally higher. However, the popularization of internet usage has awakened women’s independence, reducing their intentions to bear children. To be specific, internet usage narrows the differences between male and females in regards to resources, skills, knowledge, etc., and provides women with more job opportunities [17]. With an increase in the female labor participation rate, the traditional division of labor within the family has gradually weakened, and since the opportunity costs of reproduction being high, fertility intentions have decreased [12,18]. In any given society, changing gender roles may affect the implementation of fertility policies, which are also the causes of women’s low fertility intentions. Therefore, in China, we can reasonably believe that internet usage reduces women’s intentions to have children, to some extent; this effect can be moderated by women’s concepts of gender-based division of labor.

Based on the above considerations, this article combines the data from the Chinese General Social Survey in 2017 (CGSS2017) to systematically investigate the impact of internet usage on women’s fertility intentions. Compared to the existing literature, the marginal contributions of this article are as follows: (1) many of the previous studies are family-centered, and women with childbirth intentions and behaviors are often regarded as passive recipients of policy implementations or contraceptive services. This article overcomes these limitations and explores the theoretical mechanisms on how the frequency of internet usage affects a female’s fertility intentions. (2) Compared with Francesco (2019), who analyzed the effects of internet usage frequency and individual fertility intentions from the perspective of family–work conflicts, this article takes another approach, by introducing gender role attitudes into the research framework of women’s fertility intentions and internet usage frequency, and researches the factors affecting individual fertility intentions. (3) This work observes the problem of internet usage frequency and women’s fertility intentions in the Chinese social–cultural context, clarifies the mediating effects of gender role attitudes under the influence of internet usage frequency on women’s fertility intentions, and provides useful support for exploring solutions to the low fertility dilemma as a result of internet usage.

## 2. Materials and Methods

### 2.1. Design and Data

The data in this paper were derived from the Chinese General Social Survey in 2017 (CGSS2017), which is a national representative continuous survey project, conducted jointly by the Hong Kong University of Science and Technology and the National Survey Research Center (NSRC) at Renmin University of China. CGSS2017 covers 31 provinces (autonomous regions and municipalities) in mainland China. It adopts a multi-stage stratified random sampling method, which is conducted through individual interviews and structured questionnaire surveys. The CGSS2017 is characterized by full coverage and representativeness, and the questionnaire has good authenticity and reliability. To ensure the reliability of the empirical analysis, this paper processed the original data as follows: (1) to highlight the dominant position of women as the direct bearers of fertility intentions and fertility behaviors, we only retained the samples of women. (2) We limited the samples to women aged 18–50 years old; that is, we only kept samples of women of childbearing age. Following the exclusion of samples with missing values and invalid data resulting from refusal to answer, we finally obtained 3113 valid samples. The statistical software used in this study is STATA version 15.0 software (Stata Corp, College Station, TX, USA). The current study is approved by Chinese General Social Survey (CGSS).

### 2.2. The Basic Model

Since the dependent variable (fertility intention) in this article is a non-negative discrete random variable, which does not meet the basic assumption of a normal distribution of classical linear regression, we used Poisson regression or negative binomial regression in the counting model for parameter estimation. Assuming that female individual fertility intention *FI_i_* obeys the Poisson distribution with parameter *λ_i_*, then the conditional expectation function of *FI_i_* is:

(1) $$E\left(FI_{i}|X_{i},\beta \right)=Var\left(FI_{i}|X_{i},\beta \right)=\lambda_{i}=\exp\left({x^{\prime }}_{i}\beta \right)$$

The limitation of Poisson regression is that it requires the variance of the dependent variable to be roughly equal to the expected value; that is, “equal dispersion”. After testing the data, we found that the dependent variable did not meet the requirements of “equal dispersion”, so it was more scientific to use negative binomial regression. The usual approach is to add a term to the logarithmic expression of the conditional expectation function of Poisson regression. By introducing unobservable effects that affect women’s fertility intentions, the Poisson model can be extended as follows:

(2) $$E\left(FI_{i}|X_{i},\beta \right)=\lambda_{i}=\exp\left({x^{\prime }}_{i}\beta +\varepsilon_{i}\right)\equiv \mu_{i}v_{i}$$

where, *μ_i_* ≡ exp (${x^{\prime }}_{i}\beta$) is the deterministic function of *x_i_*, *v_i_* ≡ exp(*ε_i_*) > 0 is a random variable. Based on this, let *FI_i_* represent the women’s fertility intention and *fi_i_* still follow the Poisson distribution. Given *x_i_* and *ν_i_*, the conditional density function of *FI_i_* is as follows:

(3) P(FIi=fii|Xi,vi)=e−μivi(μivi)fiifii!     (yi=0,1,2…)

Among them, *E*(*FI_i_***|***x_i_*) = *μ_i_* = exp(*x_i_*’*β*), *x_i_* (*i* = 1,2,3,⋯) refers to various influencing factors of the fertility intention *FI_i_*. According to the setting of this article, the specific model as follows:

(4) $$\lambda_{1}=\exp\left(\varphi_{0}+\varphi_{1}Int_{i}+\varphi_{2}X_{i}+\varepsilon_{1}\right)$$

In Formula (4), *Int_i_* is an independent variable, representing the frequency of individual internet usage. *X_i_* stands for the set of control variables, including age, marital status, education level, health status, family income level, social interaction, socioeconomic status, and other variables. 

### 2.3. Selection of Variables

#### 2.3.1. Dependent Variable

This article used the individual’s ideal number of children as an indicator for the measurement of the explained variable (fertility intention). The survey item on this variable in the CGSS2017 data was: “Regardless of policy restrictions, how many children do you want to have?” This article selected this item as a substitute variable for the individual’s intention to bear children [19,20].

#### 2.3.2. Independent Variables

The independent variable of this article was the frequency of internet usage. The survey item for this variable in CGSS2017 data was “How often did you use internet media (including mobile internet access) in the past year?” Based on the respondents’ answers, internet usage was measured with a dummy variable (1 for frequently and 0 for infrequently).

#### 2.3.3. Mediating Variable

The intermediary variable in this article was gender role attitudes. CGSS2017 asked the interviewees about their views on gender role attitudes from the following two aspects: (1) “GRA1—Do you agree with the view that men put career first and women put family first?” (2) “GRA2—Do you agree that the responsibility of the husband is to make money, and the responsibility of the woman is to take care of the family?” Based on the respondents’ answers, the options ranged from “absolutely disagree” (=1), “relatively disagree” (=2), “neutral” (=3), “relatively agree” (=4), “absolutely agree” (=5).

#### 2.3.4. Control Variables

The control variables of individual characteristics were set as follows: (1) Age referred to the actual age of the interviewee. In order to test the nonlinear effect of age, we also added the age squared term. (2) Marital status was measured with a dummy variable (1 for married and 0 for unmarried). (3) The variable options of health status included “badly healthy”, “less healthy”, “normal”, “fairly healthy”, and “extremely healthy”. (4) Educational levels were expressed as “middle school and below”, “high school/vocational school”, and “college and above”. (5) The household income level was measured by the question: “What do you think of your income level in the locality?” with response categories “far below average”, “below average”, “average”, “above average”, and “much above average”. (6) As for Individual social interactions, according to the frequency of the social interactions of the interviewees, the rating ranged from “never”, “rarely”, “sometimes”, “often”, and “frequently”.

### 2.4. Mediating Effect Model

To more rigorously verify the rationality of internet usage frequency affecting fertility intentions and the mechanisms, this paper drew on Baron and Kenny [21], Iacobucci [22], and Wang, et al. [23] to use the intermediary utility test method to effectively avoid the problem of ineffective mediation. Firstly, we established a linear regression model as follows:

(5) $$FI_{i}=\beta_{1}+cInt_{i}+\gamma_{1}X_{i}+\varepsilon_{1}$$

(6) $$GRA_{i}=\beta_{2}+\alpha Int_{i}+\gamma_{2}X_{i}+\varepsilon_{2}$$

(7) $$FI_{i}=\beta_{3}+c^{\prime }Int_{i}+bGRA_{i}+\gamma_{3}X_{i}+\varepsilon_{3}$$

*Int_i_* is the dependent variable, representing the frequency of individual internet usage. *FI_i_* is the independent variable, which represents the interviewees’ fertility intentions. *GRA_i_* is the intermediary variable, and *ε*_1_–*ε*_3_ denotes the error term. *X_i_* is a set of control variables, including the interviewee’s age, marital status, education level, health status, family income level, social interaction, and socioeconomic status.

To confirm whether the significant mediation effect exists, we can test whether the null hypothesis of H_0_: *ab* = 0 holds [24]. The mediation effect can be tested via a Z-test $Z_{Mediation}=Z_{a\cdot b}/{\hat{\sigma }}_{Z_{ab}}$. We can estimate a (b) and its standard error Sa (Sb) from Equations (6) and (7). $\hat{a}$ and $\hat{b}$ are the estimation of *a* and *b*, respectively, ${\hat{S}}_{a}$ and ${\hat{S}}_{b}$ are the standard errors of $\hat{a}$ and $\hat{b}$; according to the above parameters, we can calculate $Z_{a}=\hat{a}/{\hat{S}}_{a}$, $Z_{b}=\hat{b}/{\hat{S}}_{b}$, $Z_{a\cdot b}=Z_{a}\times Z_{b}$, ${\hat{\sigma }}_{Z_{ab}}=\sqrt{Z_{a}^{2}+Z_{b}^{2}+1}$ separately, and then calculate $Z_{Mediation}=Z_{a\cdot b}/{\hat{\sigma }}_{Z_{ab}}$. Since *Z_Mediation_* is a normal distribution, so we can test the significance of the mediating effect. At the 0.05 significance level, if $\left|Z_{Mediation}\right|>1.96$, then the intermediate path is proved to be present and significant.

### 2.5. Endogenous Problems

The econometric model in this article may also have endogenous problems. Access to the internet is likely correlated with many unobservable determinants of fertility intentions (e.g., unobserved socioeconomic determinants of fertility, regional characteristics, and differences in timing preferences), which may confound the main relationship of interest; that is, whether internet usage frequency is affected by fertility intention. Another problem that needs to be solved is two-way causality. Women with higher fertility intentions have higher demands for effective parenting information, which leads to higher frequencies of network search behaviors. To address these concerns regarding endogeneity, the article employed an IV approach that exploited the average rate of internet usage across the province. The specific calculation method of this variable is that the average internet usage rate of the province is equal to the sum of the internet usage frequency of the province samples, divided by the number of province samples. The reason is that the average internet usage rate of a province has a strong correlation with the internet usage frequency of respondents. However, no evidence shows that the average internet usage rate in a province is related to an interviewee’s fertility intention. 

### 2.6. Data Analysis

A descriptive analysis using means, standard deviations, frequencies, and percentages was computed to explain the samples and variables. A negative binomial regression model was performed to verify the influence of internet usage frequency on women’s fertility intentions after controlling individual characteristic variables. We used a mediating effects model to identify the mediating effects of the gender role attitudes under the influence of internet usage frequency that affects women’s fertility intentions.

## 3. Results

### 3.1. Sample Characteristics

Table 1 shows the demographic characteristics and outcome variables of the participants. Of the 3113 participants, the average age was 36.61 years old, and the majority of the women used the internet frequently (*n* = 2179, 70.00%). The majority of women had a desire to have two children (*n* = 2087, 67.04%). The majority of participants were married (*n* = 2483, 79.67%) and reported good health status (*n* = 2085, 66.98%). A minority of the participants had a high school/vocational school (*n* = 546, 17.54%) or college degree above (*n* = 980, 31.48%).

### 3.2. Total Sample Regression Results

This article first used negative binomial regression to investigate the influence of internet usage frequency on women’s fertility intentions. The benchmark regression results are shown in Table 2. Compared with women who used the internet infrequently, women who used the internet frequently had lower intentions to give birth, and it was statistically significant (coefficient = −0.0385 *, 95%CI: −0.0680 to −0.0091). The regression results of the control variables show that higher education and fertility intentions present a significant negative correlation (coefficient = −0.0693 **, 95%CI: −0.1048 to −0.0338; coefficient = −0.0613 ***, 95%CI: −0.0942 to −0.0285), while the married women had a higher intention to bear children compared with unmarried women (coefficient = 0.1183 ***, 95%CI: 0.0714–0.1651).

### 3.3. Endogeneity Test 

To solve the problem of endogeneity, this paper applied the two-stage least square method for quantitative analysis. The results are shown in Table 3. It can be seen from the results of the first-stage test that the internet usage frequency of interviewees was highly correlated with the average internet usage rate of the province (coefficient = 0.1507 ***). The F statistic value far exceeded the threshold of the 10% bias level, which indicates that the instrumental variable is not a weak instrumental variable (T = 10.67, F = 113.77, *p* = 0.0000). The second-stage regression results showed that the frequency of internet usage had a significant negative impact on women’s fertility intentions (coefficient = −0.7223 ***, 95%CI: −1.0376 to −0.4069); this result is still robust after adding the control variables, and is basically consistent with the negative binomial regression results. Therefore, after eliminating endogeneity, the results are robust and reliable.

### 3.4. Test of Intermediary Effect of Internet Usage Frequency Influencing Fertility Intention

To analyze the mediating effects of gender role attitudes, we used stepwise regression. First, we tested the influence of internet usage frequency on women’s fertility intentions, and the results are consistent with the results in Table 2; that is, the higher the internet usage frequency, the lower the fertility intentions of women. Second, we examined the influence of internet usage frequency on the mediating variables (GRA1 and GRA2). Columns (1) and (3) in Table 4 show the results. The coefficients of the variables of internet usage frequency are all significantly negative (coefficient = −0.3270 ***, 95%CI: −0.4281 to −0.2259; coefficient = −0.2734 **, 95%CI: −0.4545 to −0.0923), indicating that the higher the frequency of internet usage, the weaker the agreement on traditional Chinese gender roles. Third, we examined the influence of both mediating variables and the frequency of internet usage variables on women’s fertility intentions. Column (2) and (4) show that the coefficients of the gender role attitudes are all significantly positive, indicating that there is a significant positive correlation between gender role attitudes and women’s fertility intentions (coefficient = 0.0288 ***, 95%CI: 0.0190–0.0386; coefficient = 0.0253 **, 95%CI: 0.0110–0.0396); that is, for women, the weaker their agreements on gender roles, the more likely they were to have fewer children. The absolute values of Z-test for variables GRA1 and GRA2 are all greater than 1.96, which indicates that the intermediate path is present and significant. The regression results in Table 4 indicates that gender role attitudes play an intermediary role in the frequency of internet usage influencing fertility intentions. For women, the higher the frequency of internet usage, the weaker the attitude of gender roles, and the lower the fertility intentions.

## 4. Discussion

Based on CGSS2017 data, this article uses negative binomial regression to verify the influence of internet usage frequency on women’s fertility intentions. The results show that the frequency of internet usage has a significant negative effect on women’s fertility intentions after controlling for other variables that might affect their fertility intentions. That is, the higher the frequency of internet usage, the lower the fertility intentions of women. China’s traditional concept of gender division of labor imprisons women more so in families, who are responsible for family production and child rearing. However, the popularization/application of the internet has brought about changes in women’s values, improving their economic status. More and more women become unwilling to play the roles of bearing and rearing children; consequently, this reduces women’s fertility intentions.

Our research combines the traditional Chinese cultural context, with particular emphasis on the mediating effects of gender role attitudes in the frequency of internet usage affecting women’s fertility intentions, confirming that the higher the frequency of internet usage, the weaker the consciousness of the traditional Chinese gender role attitudes (i.e., that “men work outside to support the family while women stay at home to take care of the family”), and the lower the fertility intentions. This suggests that individual behaviors are influenced by their values. As the theory of moral rationality put forward by Duncan and Edwards shows, individuals do not simply respond to economic or policy incentives but are impacted by their knowledge, values, and ideals [25,26]. Internet usage enables women to have more information, resources, and opportunities, which affects their fertility intentions. Moreover, the more frequently women use the internet, the more vulnerable they are to the influence of online ideological trends. This may affect their desire for fertility. Norris [27] put forward the “cultural change hypothesis” that the internet influences its participants, acting as a unique medium to influence and change the values and cultural orientations of individuals, thereby influencing individual behavioral decisions. This also provides support for our research.

The results of the control variables in econometric benchmark regression show that the higher the education levels of women, the lower the fertility intentions. According to the theory of human capital, improving education, and the abilities that empower women to directly participate in the labor market, have freed more women from raising children, allowing them to pursue their own careers and ideals. This leads to a decrease in their fertility intentions. Family economic status also has a significant impact on women’s fertility intentions. According to the theoretical model of new family economics, fertility decision-making is a function of personal preference and childcare costs under the family income limitation [28,29]. High income enables families to bear the costs of childbirth and parenting for multiple children, thereby increasing women’s fertility intentions. 

### 4.1. Implications

This article has important implications for policy-makers intending to improve women’s fertility intentions and the fertility rate. From China’s experience, increasing the female fertility rate cannot only rely on a single policy that relaxes the number of births. The negative effects of individual internet usage frequency on childbirth intentions should also be an important factor in evaluating and predicting the effects of fertility policy adjustments. In the process of formulating fertility incentive-related supporting policies, it is also necessary to optimize governance in combination with the network environment to encourage fertility intentions, to better achieve the expected effects of the fertility policy. For example, the government should build a fertility network information service platform to provide high-quality fertility information services to effectively meet women’s needs, to access parenting information and ease anxiety surrounding women’s fertility. Moreover, when investigating the influence of internet usage on fertility intentions, it is necessary to pay special attention to the roles of individual values. To increase women’s fertility intentions, it is also necessary to optimize the division of roles within the family so that women can better balance work and family. Specific measures include encouraging men to shoulder more family responsibilities, and encouraging society to bear part of the direct costs (including children’s clothing, food, housing, transportation, and medical expenses) and indirect costs (including the opportunity costs of caregivers, time costs, etc.) of raising children.

### 4.2. Limitations

The main limitations of the study are as follows: 1) this article focuses on the research surrounding women’s fertility intentions, but there may be a gap between the actual number and the ideal number of births and the fertility intentions may not necessarily be reflected in the birth behavior. However, the results of this study can still be considered valid because the intention is an important prerequisite for follow-up behavior [30,31]. Moreover, the relaxation of China’s fertility policy provides support for the transformation of fertility desire into fertility behavior. 2) This research examines the mediating effects of gender role attitudes, confirming that the weaker the traditional gender role attitudes, the lower the fertility intentions. However, awareness of gender roles cannot cover all situations. For example, participation of grandparents in parenting, as a common parenting model in China, also affects the fertility intentions of females to a certain extent. Therefore, other Chinese-specific situational factors need to be added in future research. Thirdly, at present, many scholars use artificial intelligence tools (ANN, LSTM, etc.) to conduct influence factor analyses and predictive analyses [32,33,34]. It is also of great significance to analyze the influencing factors of women’s fertility intentions and predict the changing trends surrounding the female fertility rate, which is also the research field we will pay attention to in the future.

## 5. Conclusions

Internet usage has a profound impact on personal behavior and decision-making. In China, internet usage has profoundly changed the traditional gender concept of “men work outside to support the family while women stay at home to take care of the family”, and further reduced women’s fertility intentions. How to deal with low fertility intentions caused by internet usage is a critical issue. The government should regard the negative effects of individual internet use on fertility intentions as an important consideration in evaluating and predicting the effects of fertility policy adjustments. Furthermore, future public policies need to effectively balance family role divisions between men and women, i.e., to highlight more favorable work–family balances for women.

## Figures and Tables

**Table 1:** Demographic characteristics and outcome variables of the participants (*n* = 3113).

Variable	Categories	Mean ± SD or *n* (%)
Fertility intention	0	80 (2.57)
	1	680 (21.84)
	2	2087 (67.04)
	3–10	266 (8.54)
The frequency of internet usage	Infrequently	934 (30.00)
	Frequently	2179 (70.00)
Age		36.61 ± 9.10
Marital status	Unmarried	630 (20.24)
	Married	2483 (79.76)
Health condition	Badly healthy	54 (1.73)
	Less healthy	241 (7.74)
	Normal	733 (23.55)
	Fairly healthy	1285 (41.28)
	Extremely healthy	800 (25.70)
Education level	Middle school and below	1587 (50.98)
	High school/Vocational school	546 (17.54)
	College and above	980 (31.48)
Household income level	Far below average	165 (5.30)
	Below average	1038 (33.34)
	Average	1693 (54.38)
	Above average	210 (6.75)
	Much above average	7 (0.22)
Social contact	Never	264 (8.48)
	Rarely	1016 (32.64)
	Sometimes	1089 (34.98)
	Often	636 (20.43)
	Frequently	108 (3.47)
Gender role attitudes 1	Absolutely disagree	546 (17.56)
	Relatively disagree	1071 (34.44)
	Neutral	187 (6.01)
	Relatively agree	946 (30.42)
	Absolutely agree	360 (11.58)
Gender role attitudes 2	Absolutely disagree	289 (28.00)
	Relatively disagree	269 (26.07)
	Neutral	94 (9.11)
	Relatively agree	328 (31.78)
	Absolutely agree	52 (5.04)

**Table 2:** Negative binomial regression analysis of internet usage frequency influencing fertility intention.

Variables	Coefficient	95% CI	SE
The frequency of internet usage (reference: infrequently)	−0.0385 *	−0.0680 to −0.0091	0.0150
Age	−0.0161 *	−0.0299 to −0.0022	0.0070
Age*age	0.0002 *	0.0001 to 0.0004	0.0001
Marital status(reference: unmarried)	0.1183 ***	0.0714 to 0.1651	0.0238
Health condition(reference: badly healthy)			
Less healthy	0.0420	−0.0612 to 0.1453	0.0527
Normal	0.0245	−0.0755 to 0.1246	0.0510
Fairly healthy	0.0164	−0.0834 to 0.1163	0.0509
Extremely healthy	0.0346	−0.0668 to 0.1361	0.0517
Education level (reference: Middle school and below)			
High school/Vocational school	−0.0693 ***	−0.1048 to −0.0338	0.0181
College and above	−0.0613 ***	−0.0942 to −0.0285	0.0167
Household income level (reference: far below average)			
Below average	0.0457	−0.0171 to 0.1086	0.0320
Average	0.0735 *	0.0109 to 0.1361	0.0319
Above average	0.0791 *	0.0006 to 0.1575	0.0400
Much above average	−0.0258	−0.2559 to 0.2042	0.1173
Social contact (reference: never)			
Rarely	0.0085	−0.0393 to 0.0565	0.0244
Sometimes	0.0229	−0.0247 to 0.0706	0.0243
Often	0.0431	−0.0072 to 0.0936	0.0257
Frequently	0.0046	−0.0681 to 0.0775	0.0371
Constant	0.7031 ***	0.4483 to 0.9580	0.1300
Sample size	3113		

Note: CI, confidence interval; *** Significant at *p* < 0.001. * Significant at *p* < 0.05.

**Table 3:** Endogeneity test of the influence of internet usage frequency on female fertility intention.

Variables	Coefficient	95% CI	SE
The frequency of internet usage (reference: infrequently)	−0.7223 ***	−1.0376 to 0.4069	0.1608
Age	−0.0092	−0.0368 to 0.0182	0.0140
Age*age	0.00001	−0.0003 to 0.0004	0.0002
Marital status (reference: unmarried)	0.2163 ***	0.1356 to 0.2971	0.0412
Health condition (reference: badly healthy)			
Less healthy	0.0042	−0.2184 to 0.2269	0.1136
Normal	0.0300	−0.1797 to 0.2398	0.1070
Fairly healthy	0.0579	−0.1503 to 0.2662	0.1062
Extremely healthy	0.1071	−0.1042 to 0.3185	0.1078
Education level (reference: Middle school and below)			
High school/Vocational school	0.0787	−0.0376 to 0.1950	0.0593
College and above	0.1313 *	0.0019 to 0.2606	0.0659
Household income level (reference: far below average)			
Below average	0.1320 *	0.0124 to 0.2517	0.0610
Average	0.2145 **	0.0900 to 0.3389	0.0634
Above average	0.2323 **	0.0775 to 0.3870	0.0789
Much above average	0.0162	−0.3486 to 0.3811	0.1861
Social contact (reference: never)			
Rarely	0.0253	−0.0647 to 0.1155	0.0459
Sometimes	0.0486	−0.0415 to 0.1388	0.0460
Often	0.1381 **	0.0396 to 0.2366	0.0502
Frequently	−0.0247	−0.1676 to 0.1180	0.0728
The average internet usage rate of the province	0.1507 ***	*p*-value	0.0000
Instrument variable T-value	10.67	Wald-test	110.35
F-value of the first stage	113.77	R-squared	0.1059

Note: CI, confidence interval; *** Significant at *p* < 0.001. ** Significant at *p* < 0.01. * Significant at *p* < 0.05.

**Table 4:** Test of intermediary effect of internet usage frequency influencing fertility intention.

VARIABLES	(1) GRA1	95% CI	SE	(2) FI	95% CI	SE
The frequency of internet usage (reference: infrequently)	−0.3270 ***	−0.4281 to −0.2259	0.0515	−0.0280	−0.0576 to −0.0016	0.0151
GRA1				0.0288 ***	0.0190 to 0.0386	0.0050
Constant				0.6140 ***	0.3596 to 0.8684	0.1297
Observations	3110			3110		
**VARIABLES**	**(3) GRA2**	**95% CI**	**SE**	**(4) FI**	**95% CI**	**SE**
The frequency of internet usage (reference: infrequently)	−0.2734 **	−0.4545 to −0.0923	0.0923	0.0006	−0.0567 to 0.0579	0.0292
GRA2				0.0253 **	0.0110 to 0.0396	0.0073
Constant				0.5005 *	0.0231 to 0.9780	0.2435
Observations	1032			1032		

CI, confidence interval; *** Significant at *p* < 0.001. ** Significant at *p* < 0.01. * Significant at *p* < 0.05. In order to get the standard error for the mediator variable in the regression model, we regard variables GRA1 and GRA2 as ordered variables. Z-test values for variables GRA1 and GRA2 are −4.2374 and −2.1994, respectively. Mediating test regressions were adjusted for age, Age *age, marital status, health condition, household income level, and social contact.

## Data Availability

Publicly available datasets were analyzed in this study. This data can be found here: http://www.cnsda.org/index.php?r=projects/view&id=94525591 (accessed on 5 January 2021).
